# Stability Curve Prediction of Homologous Proteins Using Temperature-Dependent Statistical Potentials

**DOI:** 10.1371/journal.pcbi.1003689

**Published:** 2014-07-17

**Authors:** Fabrizio Pucci, Marianne Rooman

**Affiliations:** Department of BioModeling, BioInformatics & BioProcesses, Université Libre de Bruxelles, Brussels, Belgium; Wake Forest University, United States of America

## Abstract

The unraveling and control of protein stability at different temperatures is a fundamental problem in biophysics that is substantially far from being quantitatively and accurately solved, as it requires a precise knowledge of the temperature dependence of amino acid interactions. In this paper we attempt to gain insight into the thermal stability of proteins by designing a tool to predict the full stability curve as a function of the temperature for a set of 45 proteins belonging to 11 homologous families, given their sequence and structure, as well as the melting temperature (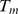) and the change in heat capacity (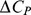) of proteins belonging to the same family. Stability curves constitute a fundamental instrument to analyze in detail the thermal stability and its relation to the thermodynamic stability, and to estimate the enthalpic and entropic contributions to the folding free energy. In summary, our approach for predicting the protein stability curves relies on temperature-dependent statistical potentials derived from three datasets of protein structures with targeted thermal stability properties. Using these potentials, the folding free energies (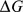) at three different temperatures were computed for each protein. The Gibbs-Helmholtz equation was then used to predict the protein's stability curve as the curve that best fits these three points. The results are quite encouraging: the standard deviations between the experimental and predicted 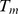 's, 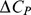 's and folding free energies at room temperature (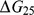) are equal to 13 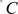, 1.3 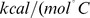) and 4.1 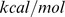, respectively, in cross-validation. The main sources of error and some further improvements and perspectives are briefly discussed.

## Author Summary

The prediction of protein stability remains one of the key goals of protein science. Despite the significant efforts of the last decades, faster and more accurate stability predictors on the proteomic-wide scale are currently demanded. The determination and control of protein stability are indeed fundamental steps on the path towards *de novo* design. In this paper we develop a method for predicting the stability curve of proteins. This curve encodes the temperature dependence of the folding free energy (). Its knowledge is important in the study of protein stability since all the thermodynamic parameters characterizing the folding transition can be extracted from it. Our prediction method is based on temperature-dependent mean force potentials and uses the tertiary structure of the target protein as well as the melting temperature () and the heat capacity change () of some other proteins belonging to the same family. From the predicted stability curves, the , the  and the  at room temperature can be inferred. The predictions obtained are compared with experimental data and show reasonable performances.

## Introduction

The understanding of the mechanisms used by nature to stabilize proteins against thermal inactivation is still an open issue of primary importance. From a theoretical perspective, such comprehension is fundamental in the study of the adaptive strategies used by the organisms to inhabit extreme environments. Due to evolution, such organisms are not only able to tolerate extreme temperature conditions, that range from less than ten degree Celsius to more than 120 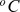, but require these conditions for their survival. The control of the thermal resistance is also important from an applicative perspective, as it would allow the optimization of a wide series of industrial, bioanalytical and pharmaceutical bioprocesses through the design and manufacture of new and more efficient enzymes [1]–[3].

In the last decades, different attempts and methods have been developed to obtain proteins of increased thermal stability. Protein engineering methods that include directed evolution methods [4]–[6] have been quite successful even if their applicability remains limited due to the intensive work required. *In silico* engineering approaches based on sequence conservation or free energy calculation methods have also been developed but with only partial success [7]–[12].

Recently, we developed a thermal stability prediction tool based on (melting)-temperature dependent statistical potentials that are derived from datasets in which only proteins with given thermostability properties are included [13]–[15]. The introduction of such potentials in the thermal stability framework is motivated by the fact that the amino acid pair interactions are temperature dependent, which means that some of them are more stabilizing than others in the high temperature regime and less stabilizing at lower temperatures (and *vice versa*) [16]–[29]. This peculiar approach allowed us to study the thermal properties of proteins without detour through their thermodynamical stability, which is advantageous since it is well known that the two types of stability are poorly correlated.

Proteins use different ways to promote their thermoresistance, which can – in a first approximation – be classified in three main strategies according to the Nojima analysis [30] (for a more recent review see also [31]). Let us start by introducing the stability curve of a protein, which can be described by the Gibbs-Helmholtz equation:
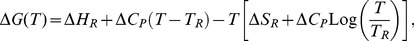
(1)where 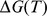 is the free energy change associated to the folding transition from the unfolded to the native state, 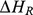 and 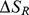 the change in enthalpy and entropy measured at the reference temperature 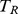, and 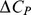 the change of the heat capacity across the transition. To obtain this equation, one has to fix the pressure of the system, to consider two-state transitions only, and to take 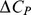 as temperature independent. Usually, the melting temperature 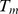, which is the midpoint of the thermal denaturation, is chosen as the reference temperature. Eq.(1) can then be rewritten as:
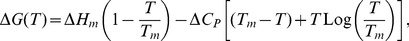
(2)where 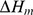 is the enthalpy measured at 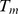. Sometimes, the reference temperature is taken equal to 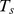, the temperature of maximal stability, which yields the equation: 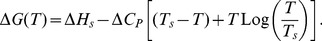
(3)


**Figure 1:**
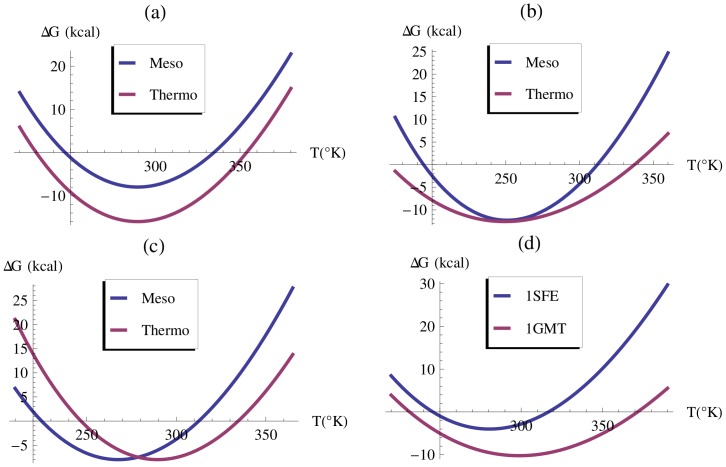
Stability curves of thermostable and mesostable proteins. (a,b,c) Different strategies of thermal adaptation of hypothetic proteins. (d) Comparison between the stability curve of *Tk*-MGMT (PDB [47] code 1GMT) and its mesophilic counterpart *Ec*-AdaC (PDB code 1SFE) [32].)

The first strategy that a protein can use to increase its thermostability [30] is to make the enthalpy change (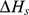) measured at 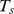 more negative. This yields an overall decrease of 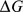 for all temperatures as we can see from Eq.(3) (Figure 1.a). In the second strategy, 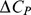 becomes less negative, which leads to an increase of 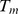 through a modification of the shape of the curve (see Eq.(2) and Figure 1.b). The last strategy consists in an increase of the maximum stability temperature, 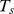, defined at the minimum of the 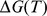 curve, where the transition is purely enthalpic. This shifts the curve towards the high temperature region (see Figure 1.c).

It is, in general, not obvious to determine which type of strategy is adopted by a given protein; often several strategies are used in combination [31]. A realistic example of stability curve is depicted in Figure 1.d: the value of the folding free energy 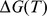 is plotted both for a thermostable protein, the 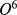 -methyl-guanine-DNA methyltransferase from *Thermococcus kodakaraensis* (*Tk*-MGMT) with 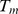  = 98.6 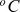, and for its mesostable counterpart, the C-terminal Ada protein from *Escherichia coli* (*Ec*-AdaC) with 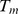  = 54.8 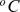, as determined experimentally in [32]. We can clearly see that in this case the three strategies are used simultaneously in the achievement of a higher thermal stability.

The strategies for improving the thermal resistance of a protein sometimes also improve the thermodynamic stability, defined by the folding free energy 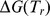 at room temperature (25 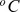), and sometimes not. The first strategy clearly does; for the other two strategies, it depends on the relative values of 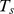 and 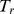 (see Figures 1a–c).

It is unfortunately quite difficult to get accurate predictions of thermal stability. The results described in the literature are in general family-dependent and sometimes even contradictory [16]–[29]. Indeed, the temperature-dependent nature of the amino acid interactions makes the thermal stability analyses quite intricate and the mechanism behind it difficult to unravel. Predicting the thermodynamic stability is not easy either. There are no methods for predicting the thermodynamic stability of a given protein, with the notable exception of molecular dynamic simulations, which are however very time-consuming and not applicable on a large or medium scale. Only methods for predicting thermodynamic stability changes upon point mutations (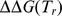) have been developed and reach good scores [33]–[43]. No predictions of the enthalpy 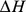 or entropy 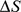 do exist either. In contrast, the prediction of 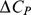 is relatively easy since it is strongly correlated to the change of accessible surface area upon unfolding [44]–[46].

In this paper we go a step further than previous analyses aiming at evaluating either 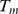, 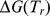 or 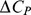. We indeed present a method for predicting the whole stability curve 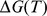 of a protein from its sequence and structure, in the temperature range that is relevant for such systems (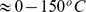), using as main tool the temperature-dependent statistical potentials developed and tested in [13]. We would like to emphasize that this is, to our knowledge, the first prediction method that outputs the complete stability curve. To get a satisfactory performance, we used in the predictions some information about proteins belonging to the same homologous family, and more precisely their 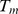 and 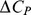. The predicted stability curve yields an estimation of the melting temperature 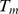, the thermodynamic stability 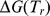, the temperature of optimal stability 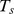, the 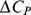, as well as the enthalpy 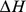 and the entropy 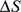 at certain temperatures. We present our results in cross validation for a set of 45 proteins belonging to eleven homologous families (for the list of their PDB codes [47] and their characteristics, see Table S1 of Supporting Material). The predicted values are compared with the experimentally determined values when available, and the different strategies used by the proteins for thermal stabilization are investigated and discussed.

## Methods

### 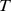-dependent statistical potentials

**Figure 2:**
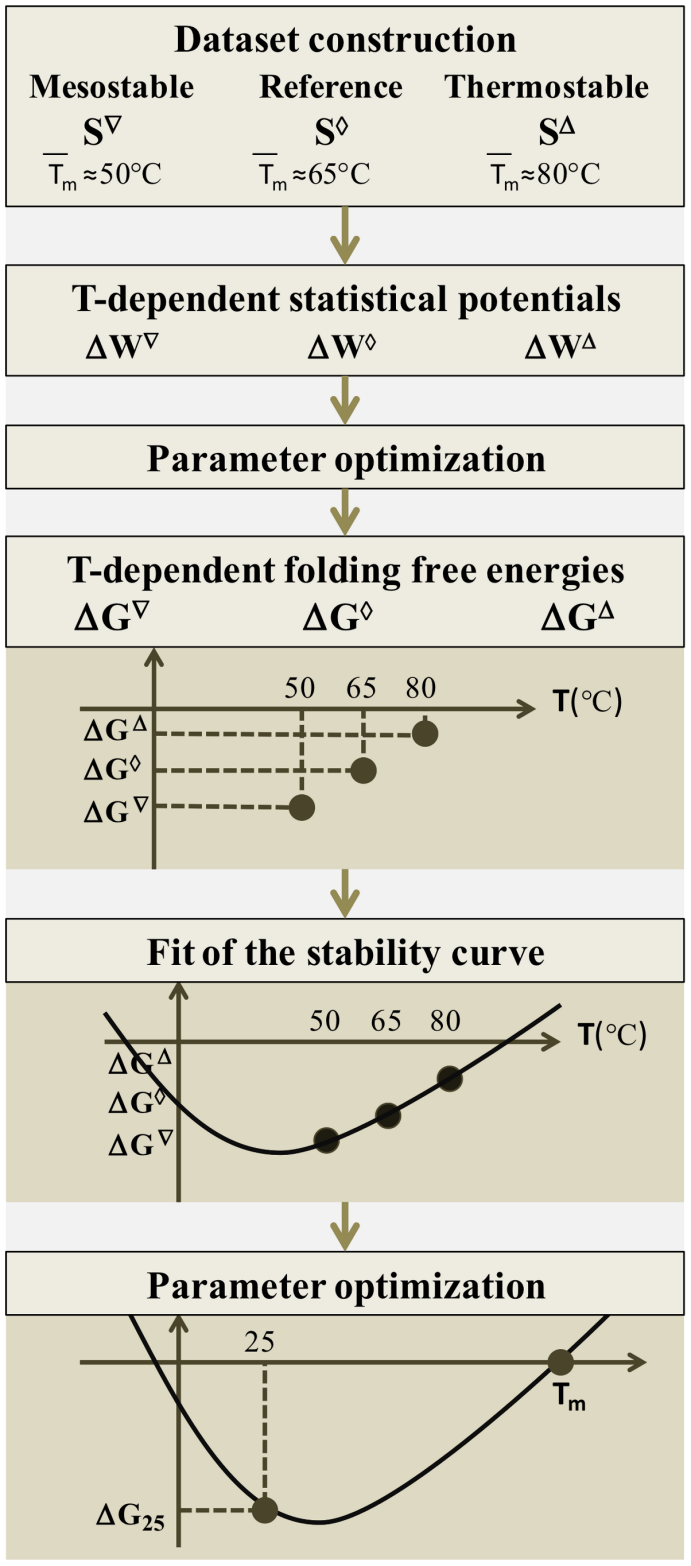
Flowchart of the protein stability curve prediction method.

In this section we describe the main tools used in this analysis, namely the statistical potentials, and how they have been optimized for the current investigation. The main steps of our approach are schematically illustrated in Figure 2.

The statistical potentials are well known since some seminal papers [48]–[50]. They are derived from the frequency of associations between certain sequence and structure elements in a dataset of experimentally determined native protein structures. Even though such potentials have been extensively and successfully used in the analysis of the thermodynamic stability of proteins, they have only recently been applied in the thermal stability context, where the temperature dependence of the amino acid interactions must be taken into account [13]–[15]. To deal with this, potentials that depend on the melting temperature were derived from different datasets in which only proteins with given thermal properties were included. Three such datasets were considered [15]: a set containing only mesostable proteins, denoted 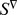 and characterized by a mean value of the melting temperature of its entries (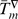) of about 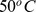, a thermostable ensemble, denoted 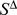, with 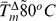, and a reference set containing both mesostable and thermostable proteins, denoted 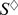, with 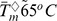. The list of proteins belonging to these datasets are given in Table S0–S11 and Table S13 of the Supplementary Material of [15].

From these different datasets, statistical potentials were derived using the standard formalism of the inverse Boltzmann law [13], [14]:
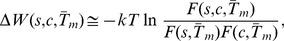
(4)where 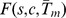 is the relative frequency of observation of the sequence element 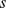 associated to the structure element 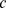, and 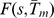 and 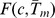 are the frequencies of observation of the sequence element 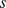 and of the structure element 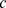, respectively. In this computation, 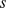 corresponds either to the amino acid type 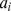 of residue 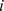 along the polypeptide chain, or to the amino acid types 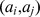 of residues 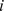 and 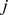, while 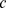 is either the backbone torsion angle domain 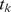 of residue 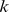, as defined in [51], or the spatial distance 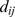 between the residues 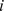 and 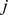. The former are called torsion potentials and the latter distance potentials.

While the torsion potentials describe local interactions along the chain and are a measure of the propensity of a given amino acid to adopt certain backbone torsion angles, the distance potentials describe the tertiary interactions and measure the propensity of amino acids to be separated by a certain spatial distance 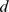. The values of the distance between two residues, defined as the distance between the geometrical centers of the heavy side chain atoms, range between 3.0 and 8.0 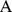 and were grouped into 25 bins of 0.2 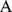 width, with two additional bins that contain distances larger than 8.0 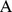 and smaller than 3.0 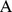, respectively.

Note that we have made the 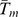 -dependence of the frequencies explicit to stress that these are computed from a dataset associated with specific thermal properties, characterized by 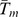. As a consequence, the potentials are 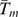 -dependent and reflect the thermal characteristics of the dataset from which they are derived.

Due to the smallness of the dataset, some techniques are required to smooth the potentials and improve their performances. A first modification that has been performed is a correction for sparse data consisting in rewriting the frequencies as [52]:
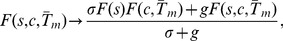
(5)where 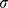 is an adjustable parameter chosen to be equal to 10 for the distance potentials and to 20 for the torsion potentials (based on preliminary tests), and where 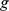 is equal to 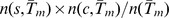. This correction ensures that the potentials tend to zero when the number of observations in the data set is too small. A second trick that has been used consists, for a given bin 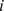, in summing the number of occurrences of the neighboring bins giving them a decreasing weight: 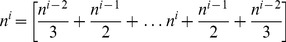
(6)where 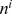 is the number of occurrences in bin 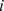.

### Families of homologous proteins

Predicting the stability curve of proteins from their sequence and structure alone is quite a difficult task. To slightly simplify the problem, we focused on families of homologous proteins, and make predictions that take into account some informations from the other family members. We therefore searched the full protein set 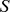 for families of homologous proteins with at least three members of known 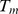. We found 11 such families containing both mesostable and thermostable proteins. They are: 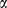 -amylase, acylphosphatase, lysozyme, myoglobin, 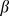 -lactamase, 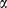 -lactalbumin, adenylate kinase, cell 12A endoglucanase, cold shock protein, cytochrome P450 and ribonuclease. The complete list of the 45 proteins belonging to these families is given in Table S1 of Supporting Material.

Some quantities (such as the number of residues, 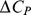, etc.) remain approximately constant inside a given family. This obviously makes the prediction method simpler to build. Such family-dependent analysis remains nevertheless quite intricate, since the thermostability properties of the proteins of a given family are sometimes very different.

In order to improve the performance of our method, the datasets 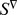, 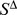 and 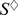 have been further enlarged by adding proteins that belong to the protein family considered but whose 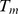 was estimated from their environmental temperature instead of being experimentally determined; note that the pairwise sequence identity within each set was kept below 25% to avoid biasing the potentials (see [15] for details about the dataset construction procedure). Strictly speaking, this modification makes the datasets and the corresponding potentials family dependent.

### Computation of the folding free energy at different temperatures

The folding free energy 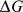 of a given protein is computed at the temperatures 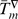, 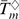 and 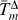 from the (melting-)temperature dependent potentials defined in the previous subsections. More precisely, we have:
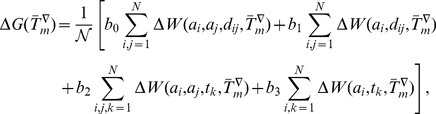
(7)

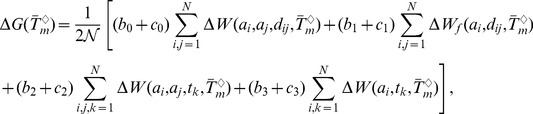
(8)

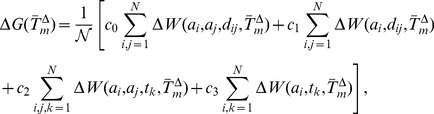
(9)where 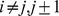 for the distance potentials, 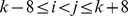 for the torsion potentials, and the parameters 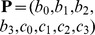 are positive real numbers. The normalization coefficient 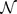 is defined as:
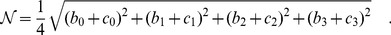
(10)


The temperatures (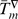, 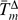, 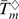) correspond to the average melting temperatures of the mesostable, thermostable and average datasets. The real 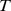 -dependence of the folding free energies is obviously related to these melting temperatures. However, it would be a very strong (and obviously wrong) assumption to suppose that the average melting temperatures and the real temperatures are equal. Rather, as will be seen in the next subsection, a scale parameter must be introduced to relate the 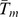 's to the real 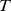.

The strategy for identifying the parameter values 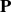 consists in maximizing the anticorrelation between the melting temperature and the difference in free energies 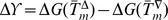. Indeed, 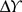 has been shown to be much more correlated to the melting temperature than the folding free energy 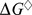 [15]. The optimization is performed on all proteins with known 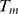 (listed in Table S1), excluding those of the protein family 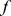 that we want to predict:
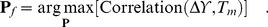
(11)


The subscript 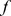 indicates the family-dependent nature of the coefficients since their optimization is performed without the proteins of 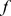. This avoids the overestimation of the performance, and amounts to cross validation. All the optimizations described in this paper are performed using the ordinary least square regression method implemented in *Mathemetica* 7.0.

### Extrapolation of the full stability curve

In the next steps of the computation, we estimate the full stability curve given by Eq.(2) from the three values of the folding free energies given by Eqs(7–9), for the set of 45 proteins from the 11 protein families. Let us assume for the moment that the 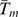 -dependence is the true 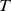 -dependence of the potentials. Under this assumption, the stability curve can easily be obtained: it is has the form (2) and depends on the thermodynamic quantities (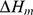, 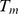 and 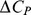), viewed as parameters, which are identified to best fit the three data points:
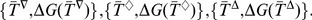
(12)


However, this simple approach does not give accurate predictions, both because the 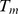 - and 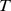 -dependences differ and because the error on these three points, which are moreover quite close along the 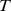 -axis, leads to large errors on the whole curve. Three different issues must be solved to get reasonable stability curves.

The first issue concerns the sign of the second derivative of the curve. In a few cases (less than 10%), this sign is wrong, which implies that the curve is upside-down and the protein seems unfolded in the physiological temperature range. This error is related to the fact that the three points given in Eq.(12) are too close along the 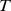 axis; this is due to the limited number of known proteins with very low or very high 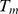. The shape of the curve depends thus strongly on the relative position of the average point 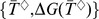 relative to the mesostable and thermostable points 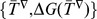 and 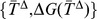. Sometimes even a small variation of these values can lead to the inversion of the shape of the curve.

To overcome this problem, we imposed a fourth point in the fitting procedure, in addition to those given in Eq.(12). This point is taken at a temperature of 0°K, where we impose 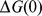 to be equal to the average of the 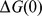 's of the other proteins that belong to the same family. This quantity has no physical interpretation, as the inverse bell shape of the stability curve may not be extrapolated to zero temperature; indeed, we have in reality 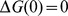. This trick is however quite useful to impose the correct sign of the second derivative of the curve in the physiological temperature range.

This procedure has been applied when the predicted curve is upside-down, but also when the value of 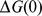 deviates by more than one standard deviation from the mean 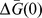 computed inside the family 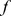. This leads to an overall improvement of the results since it smooths out possible errors on the average point 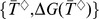, which is amplified in the curve derivation procedure.

The second issue is the determination of the overall scaling factor 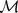 of the curve. When more than one value of 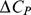 was experimentally determined within the considered family 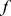, we fix 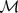 for the protein 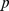 in the family 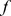 as the ratio:
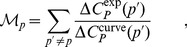
(13)where 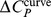 is extracted from the predicted curves as the coefficient of the 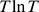 term, 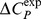 is the experimental value and the sum is over the proteins belonging to 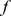 excluding 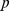; this again amounts to obtain predictions in cross validation. If only one or no 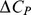 values were available for the family, we took as normalization factor the mean of the 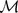 values found for the other families, excluding the largest and smallest values. This is a rough approximation since this quantity is expected to be strongly family dependent. However, despite the crude approximations made, the final result shows a fair performance that will certainly improve when more data or an independent 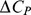 determination will be available.

The last issue concerns the real temperature dependence of the potentials. Strictly speaking, the 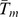 -dependence of the potentials is different from the real 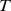 -dependence, even though they are obviously related. Indeed, the temperature resistant interactions can be expected to play a fundamental role in the stabilization in the high temperature regime and *vice versa* in the low temperature region (see [16]–[20] for the temperature dependence of the amino acid interactions). The assumption that we made is that the real 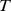 value at which the potentials are calculated is related to the value of 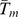 by a multiplicative factor that we call 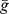, which is assumed to be different for each protein. The strategy for fixing it is the following: once the function 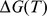 has been estimated for all the proteins 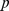 of a given family 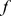, we determined the temperature 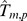 at which it is zero. We identified 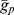 for a protein 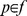 so as to minimize the cost function:
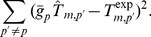
(14)


Since we are working in cross validation, the sum is over the proteins 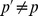 that belong to family 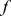. For a given protein 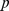, the folding free energy is thus given as 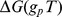.

## Results

The prediction of the mechanisms used by proteins to enhance their thermoresistance is a highly non-trivial issue. The principal mechanisms of this stabilization can be schematically described in terms of three strategies (see Figures 1a–c). The first consists in a global decrease of the folding free energy 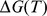 at all temperatures, which automatically implies an increase of the melting temperature. The second strategy consists of less negative values of 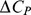, which broadens the stability curve. In the third strategy the temperature of maximal stability 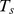 undergoes a shift towards the high temperature region. It is not simple to understand which mechanism is used by each protein and if it is used alone or in combination [31]. Moreover, different proteins of the same family can reach higher thermostability through completely different mechanisms.

**Figure 3:**
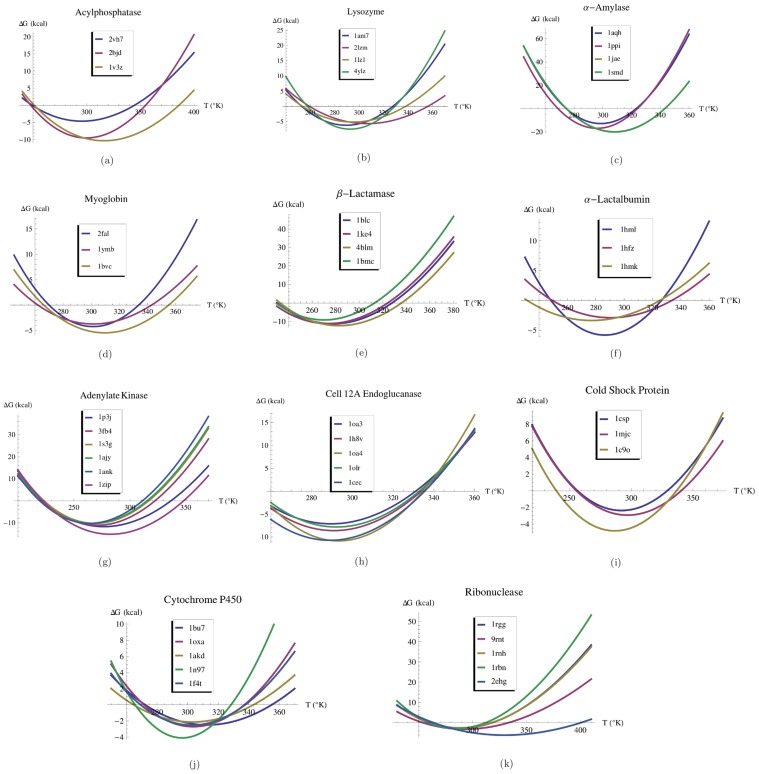
Predicted stability curves of the 45 proteins considered, which belong to 11 homologous families. The PDB codes, the host organisms and their environmental temperatures of all the proteins are given in the following list: (**a**) 2vh7 (*Homo sapiens*, 37 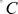), 2bjd (*Sulfolobus solfataricus*, 80 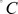), 1v3z (*Pyrococcus horikoshii*, 98 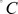). (**b**) 1am7 (*Bacteriophage lambda*, 37 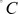), 2lzm (*Escherichia coli*, 37 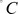), 1lz1 (*Homo sapiens*, 37 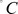), 1am7 (*Gallus gallus*, 41 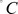). (**c**) 1aqh (*Alteromonas haloplanktis*, 26 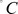), 1ppi (*Sus scrofa*, 39 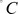), 1jae (*Tenebrio molitor*, 28 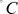), 1smd (*Homo sapiens*, 37 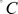). (**d**) 2fal (*Aplysia limacina*, 17 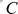), 1ymb (*Equus caballus*, 38 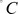), 1bvc (*Physeter catodon*, 35 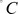). (**e**) 1blc (*Staphylococcus aureus*, 34 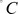), 1ke4 (*Escherichia coli*, 37 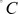), 4blm (*Bacillus licheniformis*, 43 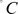), 1bmc (*Bacillus cereus*, 30 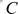). (**f**) 1hml (*Homo sapiens*, 37 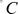), 1hfz (*Bos taurus*, 38 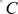), 1hmk(*Capra hircus*, 39 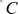). (**g**) 1p3j (*Bacillus subtilis*, 37 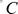), 3fb4 (*Jeotgalibacillus marinus*, 18 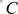), 1s3g (*Bacillus globisporus*, 15 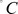), 1aky (*Saccharomyces cerevisiae*, 28 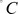), 1ank (*Escherichia coli*, 37 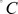), 1zip (*Bacillus stearothermophilus*, 51 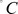). (**h**) 1oa3 (*Hypocrea schweinitzii*, 40 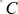), 1h8v (*Thrichoderma reesei*, 35 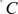), 1oa4 (*Streptomyces sp. 11ag8*, 30 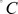), 1olr (*Humicola grisea*, 50 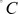), 1cec (*Clostridium thermocellum*, 60 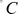). (**i**) 1csp (*Bacillus subtilis*, 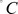), 1mjc (*Escherichia coli*, 37 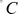), 1c9o (*Bacillus caldolyticus*, 70 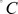). (**j**) 1bu7 (*Bacillus megaterium*, 30 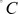), 1oxa (*Saccharopolyspora erythraea*, 31 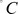), 1akd (*Pseudomonas putida*, 30 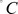), 1n97 (*Thermus thermophilus*, 68 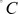), 1f4t (*Sulfolobus solfataricus*, 78 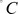). (**k**) 1rgg (*Streptomyces aureofaciens*, 28 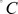), 9rnt (*Aspergillus Oryzae*, 49 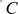), 1rnh (*Escherichia coli*, 37 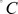), 1rbn (*Bos taurus*, 38 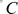), 2ehg (*Sulfolobus tokodaii*, 80 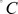).

In order to gain understanding into the thermal stability enhancement strategies and to obtain some quantitative predictions, we designed a method to predict the full stability curve of 45 proteins that belong to 11 homologous families (see Methods section). The results are the 45 stability curves given explicitly in Table S3 and plotted in Figure 3.

To make the analysis quantitative, we extracted from these predicted stability curves three independent thermodynamic parameters that define the transition, namely 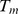, 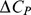 and 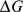 at 25 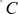, and compared them with the experimental values. For the melting temperature, the experimental values are known for all 45 entries while for the other two quantities, they are known for 17 and 16 proteins, respectively (see Table S2). We report in Table 1 the standard deviation between the computed and the experimental values, as well as the correlation coefficient between the two quantities with the corresponding P-values.

**Table 1:** Standard deviation (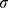) and linear correlation coefficient (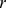) between the experimental and predicted thermal and thermodynamic parameters.

Parameter	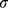	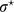	r	r 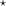	N (N 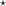 )	P-value
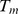	13.4  C	10.2  C	0.69	0.76	45 (40)	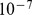
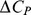	1.3 kcal/(mol  C)	0.7 kcal/(mol  C)	0.92	0.41	17 (15)	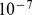
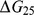	4.1 kcal/(mol)	2.6 kcal/(mol)	0.42	0.69	16 (14)	0.05

1 In the computation of 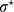 and 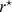, the 10% worst predicted proteins are excluded. N is the number of proteins for which experimental data are available and the results are computed.

Let us start with the analysis of the melting temperature whose values are simply extracted from the protein stability curves 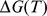 by looking for the zero of Eq. (2), since by definition:
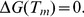
(15)


**Figure 4:**
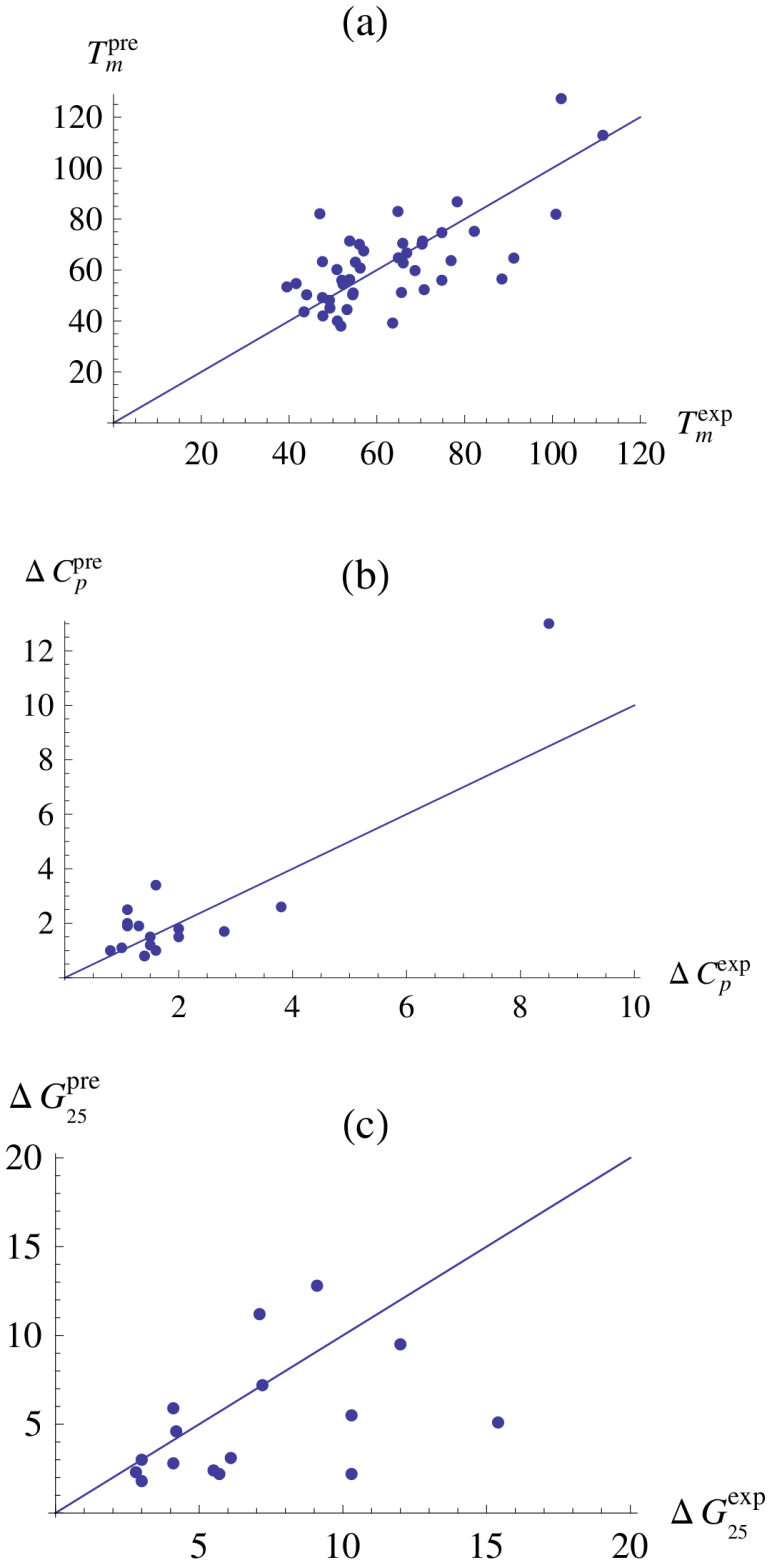
Comparison between: (a) the experimental and predicted melting temperatures (in °C), (b) the experimental and computed 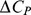 (in kcal/(mol °C)) and (c) the experimental and the predicted 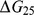 (in kcal/mol), for the set of 45 proteins belonging to the 11 homologous families. The straight lines correspond to the bisector of the first quadrant (y = x).

The value of the standard deviation between the experimental and the so computed 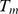 's is, in cross validation, equal to about 13 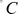 and reduces to 10 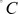 when the 10% worst predicted entries are excluded (Table 1). This value is comparable with the one found previously with a different method [15], with the notable difference that we predict here simultaneously the whole stability curve. In Figure 4.a, the predicted versus the experimental 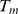 's are plotted; the corresponding correlation coefficient 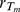 is found to be equal to 0.69 (P-value 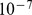), and to increase to 0.76 upon exclusion of the 10% worst predicted proteins.

We also computed the 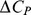 for all the proteins belonging to the eleven homologous families. In this prediction, the identification of the normalization factor 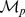 defined in Eq. (13) is fundamental. Unfortunately, we do not have enough input data, *i.e.* experimental 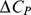 's, to identify this parameter inside each family: only for 17 entries is the 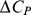 known, with moreover often quite large experimental errors (of the order of 10–20%). When performing predictions in cross-validation, we have thus to fix 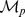 independently of the other proteins of the family (using the procedure explained in Methods) for more than half of the entries, which inevitably gives rise the errors.

The standard deviation between the experimental and the predicted values of 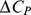 is reported in Table 1. It is equal to 1.3 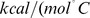) and reduces to 0.8 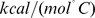 when the two worst predicted proteins are excluded. The experimental and predicted values are plotted in Figure 4.b; the correlation 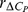 between the two quantities is equal to 0.92 (P-value 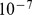), but falls down to 0.41 upon exclusion of the two worst predictions.

We chose as last independent quantity that can be extracted from the predicted curves the folding free energy at 25 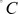 (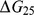). The considerations made in the previous paragraph about the normalization factor 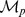 are valid for this quantity too and thus we cannot expect a perfect correlation between the predicted and experimental values due to the lack of data. We found indeed a standard deviation of 4.1 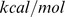 between predicted and measured 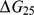 's, which reduces to 2.6 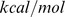 when the two worst predicted proteins are excluded. The correlation coefficient 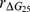 between the experimental and the predicted values is 0.4 (P-value 0.05) and 0.7 upon exclusion of the two worst predictions. These results are shown in Table 1 and plotted in Figure 4.c. A list of values of 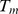, 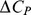, and 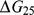 predicted from the 45 stability curves, as well as the corresponding experimental values where available, are reported in Table S2 of Supporting Material.

A further outcome that can be derived from the predicted stability curves is a better understanding of the strategies used within each protein family to reach a higher thermal stability. In particular, we can evaluate quantitatively the correlation between the thermodynamic and thermal stabilities: the linear anticorrelation between 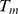 and 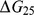 (usually taken as the descriptor of the thermodynamic stability) is relatively high and is of the order of 0.7 when the two worst predicted families are excluded. The increase of the thermodynamic stability thus remains the principal mechanism for the thermal stability enhancement. The reason for this is that single amino acid substitutions can cause much easier an increase of the number of thermodynamically stabilizing interactions, such as hydrogen bonds and hydrophobic interactions, than for example a shift of the optimal stability temperature 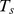 towards higher 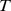, for which more complex amino acid substitutions are in general necessary. This result, which has already been obtained on the basis of experimental data [31], [53], is here derived purely on the basis of our predictions.

The other two mechanisms for enhancing the thermostability, discussed in the previous sections, turn out to be important too even though they show a lower correlation with the melting temperature. In particular, the shift of the maximum stability temperature 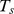 has a linear correlation coefficient of about 0.5 with 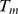 and the change in heat capacity 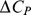 an anticorrelation coefficient of about 0.3, when excluding the two worst predicted families.

These predicted values can be compared with experimental data for the few proteins for which the full stability curve has been determined and thus similar correlation coefficients between 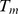 and 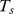, and between 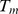 and 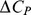 can be computed (see for example [53]). Notably, the experimental correlation coefficients 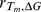 and 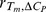 are equal to 0.6 and 0.2, respectively, and are thus quite close to the correlation coefficient predicted by our method. The shift of 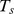 towards higher 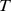 appears thus to be a preferred method for enhancing the thermostability compared to the change in 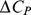. In other words, the reduction of the conformational entropy in the denaturated state or its increase in the native state seems easier to achieve compared to a change of 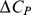.

## Discussion

The full understanding of protein thermal stability remains a challenge in protein science despite the large amount of research on this topic the last decades. As a matter of fact, it is globally more intricate to understand than the thermodynamic stability. Indeed, besides the problem due to the marginal stabilization achieved by a delicate balance of opposite forces, it poses the additional – and not the least – issue of the temperature dependence of the amino acid interactions, which is barely known.

We have designed a method based on (melting)temperature-dependent statistical potentials to deepen the thermal stability investigation. The basic idea behind this approach is simple and consists in constructing different datasets in which only proteins with given thermal properties were considered. Mean force potentials were extracted from sequence-structure frequencies computed from these datasets, following the standard statistical potential formalism, and hence reflect their thermal characteristics. They actually represent the amino acid interactions at some temperature that is related to the average 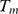 of the proteins in the dataset. The folding free energy of a given protein at a given temperature was estimated on the basis of these 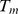 -dependent potentials. More precisely, three different datasets with different average 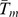 's were constructed, from which three folding free energies at these 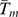 's were computed for each protein. The identification of the protein's full stability curve was accomplished by the identification of the modified Gibbs-Helmholtz equation (2) that best fits these three points.

Before concluding with future perspectives, let us summarize briefly the performance of the method and the main errors that affect it. The standard deviations between the experimental and computed quantities are equal, in cross-validation, to 13 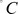, 1.3 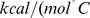) and 4.0 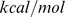 for the melting temperature, the 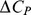 and the folding free energy at 25 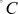, respectively. These results can be considered as rather good especially if one considers the three main sources of error that we have encountered. The first source is certainly the lack of data. As already stressed in the main text and in [15], we do not have enough experimentally resolved proteins with known 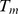 to build larger datasets and thus more accurate potentials, even though we introduced some tricks to partly overcome this problem. This issue will certainly be improved when more experimental data will be available. The second source of error is related to the presence of ligands in some of the analyzed families, which contribute strongly to the protein stabilization but which we unfortunately cannot take into account with our statistical potentials. Finally, the measurement errors are sometimes quite significant, especially due to the fact that the experiments are not performed exactly in the same environmental conditions. These different issues taken together significantly increase the error on the predictions.

A noteworthy result that can be deduced from our predictive approach is that the preferred mechanism for enhancing the thermostability is an increase of the thermodynamic stability, in agreement with previous results based on experimental data [31]. Unfortunately, this does not allow us to construct an accurate predictor for the melting temperature on the basis of the thermodynamic stability only [15], since the other thermostabilizing mechanisms turned out to be important too – although to a lesser extent. Taking these other mechanisms into consideration as we did in this paper led us to a prediction method with much better performances, which we moreover hope to further improve in the near future. Furthermore, the analysis of the thermal stability optimization strategies has also shown that it is not possible to determine a unique molecular cause or a thermodynamic effect that explains the complexity of the thermal resistance modulation for the different families, since different strategies are used in combination.

We would like to underline the main strength of our approach that is the possibility to predict at once all the thermodynamic parameters that characterize the protein folding transition. We can indeed predict with our method both the thermodynamic and thermal stabilities in a large temperature range. As far as we know this is the only method that is able to do that, and moreover it does so in a fast and relatively accurate way. Neither the standard statistical potential formalism nor the molecular dynamics simulations or the coarse-grained computational approaches to protein folding are able to consider explicitly the temperature dependence of the amino acid interactions and give predictions for both kinds of stabilities.

However, some points of the present analysis can still be improved, and we plan to do so in a future investigation. In particular, we will try to supply to the lack of data by enlarging the dataset of proteins whose thermal properties have been measured experimentally and subdivide it in more than three subsets so as to be able to get more reliable fits of the stability curves.

Two different ways can be explored to enlarge the datasets. The first consists in adding proteins with known structure but unknown melting temperature. To decide to which of the thermal ensembles these additional proteins belong, one could estimate their 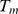 from the method presented in this paper or from the environmental temperature of their host organism. The other strategy consists in the use of proteins with known melting temperature, whose structures are unknown but could be obtained by comparative modeling techniques. This approach is motivated by earlier analyses that tested modeled structures for the prediction of thermodynamic stability changes upon point mutations on the basis of standard statistical potentials [54]. Indeed, predictions applied on modeled structures have been shown to undergo a surprisingly small accuracy loss compared to experimental structures owing to the coarse-grained structural representation on which the potentials are based. This finding lets foresee an increase of the overall accuracy of our 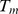 prediction method due to the enrichment of the datasets with modeled structures. But it also foreshadows the applicability of the resulting prediction method to low-resolution or modeled structures, with good performances. This undoubtedly increases the potentialities and interest of our approach.

We expect the enlargement of the datasets to play an important role in the reduction of the prediction errors, since it will allow us to define more than three datasets and thus to compute the folding free energies of a target protein at more than three different temperatures. This should definitely reduce the consequence of the errors on the predicted points in the 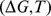 -plane when fitting the stability curve through those points. Moreover, larger datasets will allow us to consider more types of statistical potentials (for example potentials that depend simultaneously on amino acid types, interresidue distances and backbone torsion angle domains [52]), which are now forbidden for statistical significance reasons.

Note finally that the current version of our prediction method is family-dependent, as the datasets vary slightly from one family to another and the optimization of some parameters is performed inside the families (see Methods section). We would like to stress that this procedure does in no way bias the predictions. All our tests are indeed performed in pure cross validation. Rather, this procedure improves the predictions by exploiting relevant information that characterizes the homologous families. Another promising improvement of our prediction method, which would make it applicable to any target protein of known structure, consists in extending the current version without too much accuracy loss to the more general case that ignores any reference to homologous proteins.

In conclusion, although there is still room for improvements and generalizations, our approach has opened a novel and original way for designing fast and accurate predictors of protein stability at different temperatures.

## Supporting Information

Table S1 List of 45 proteins with known melting temperature analyzed in this study.
(PDF)

Table S2 Predicted and experimental values of the thermodynamic and thermal parameters for the set of 45 proteins.
(PDF)

Table S3 Analytic expression of the predicted stability curves (in kcal/mol) for the set of 45 proteins.
(PDF)
