# Supplementary material for: Stability Curve Prediction of Homologous Proteins Using Temperature-Dependent Statistical Potentials
Source: PLoS Comput Biol. 2014 Jul 17;10(7):e1003689. doi: 10.1371/journal.pcbi.1003689 (PMC4102405; doi:10.1371/journal.pcbi.1003689)
Supplement: Table S1 — List of 45 proteins with known melting temperature analyzed in this study. (PDF) [file pcbi.1003689.s001.pdf]

Table S 1: List of 45 proteins with known melting temperature analyzed in this study.

**Protein** PDB code	T_m^{exp} (°C)	pH	Protein Name	Res. ($\mathring{A}$)	Host Organism	T_{env} (°C)	N	Reference
1aqh	43.7	7.2	α-amylase	2.00	*Alteromonas haloplanctis*	26.0	448	J Biol Chem 276, 25791 (2001)
1ppi	65.6	7.2	α-amylase	2.20	*Sus Scrofa*	39.0	496	J Biol Chem 276, 25791 (2001)
1jae	65.9	7.2	α-amylase	1.65	*Tenebrio Molitor*	28.0	470	J Biol Chem 276, 25791 (2001)
1smd	70.3	7.2	α-amylase	1.60	*Homo Sapiens*	37.0	495	J Biol Chem 276, 25791 (2001)
1am7	52.3	7.0	Lysozyme	2.30	*Bacteriophage Lambda*	37.0	150	FEBS Lett 460, 442 (1999)
2lzm	64.8	6.5	Lysozyme	1.70	*Escherichia Coli*	37.0	164	Nature 334,406 (1988), PNAS 85,401 (1988)
1lz1	64.9	2.7	Lysozyme	1.50	*Homo Sapiens*	37.0	130	J Mol Biol 254, 62 (1995)
4lyz	74.8	7.0	Lysozyme	2.00	*Gallus Gallus*	41.0	129	Proteins 40, 49 (2000)
2fal	52.0	7.0	Myoglobin	1.80	*Aplysia Limacina*	17.0	146	J Mol Biol 297, 1231 (2000)
1ymb	78.3	7.0	Myoglobin	1.90	*Equus Caballus*	38.0	153	Biochemestry 4, 5075 (2001)
1bvc	82.2	9.6	Myoglobin	1.50	*Physeter Catodon*	35.0	153	Protein Sci 2, 1099 (1993)
1blc	41.6	7.50	β-lactamase	2.20	*Staphylococcus Aureus*	34.0	257	Biochemestry 33, 116 (1994)
1ke4	54.6	6.80	β-lactamase	1.72	*Escherichia Coli*	37.0	357	Protein Sci 8, 1816 (1999)
4blm	66.0	7.00	β-lactamase	2.00	*Bacillus Licheniformis*	43.0	256	Biochemestry 29, 5797 (1990)
1bmc	51.0	7.00	β-lactamase	2.50	*Bacillus Cereus*	30.0	213	Biochemestry 61, 6603 (1992)
1hml	39.5	7.00	α-lactalbumin	1.70	*Homo Sapiens*	37.0	123	Biochemestry 28, 8568 (1989)
1hfz	56.2	7.40	α-lactalbumin	2.30	*Bos Taurus*	38.0	123	Protein Eng 12, 581 (1999)
1hmk	70.8	7.50	α-lactalbumin	2.00	*Capra Hircus*	39.0	121	Proteins 60, 118 (2005)
2vh7	53.8	5.50	Acylphosphatase	1.45	*Homo Sapiens*	37.0	99	Proteins 62, 64 (2006)
2bjd	100.8	5.50	Acylphosphatase	1.27	*Sulfolobus Solfataricus*	80.0	90	Proteins 62, 64 (2006)
1v3z	111.5	7.40	Acylphosphatase	1.72	*Pyrococcus Horikoshii*	98.0	91	Biochemestry 38, 33 (2005)
1p3j	47.6	7.40	Adenylate kinase	1.90	*Bacillus Subtilis*	37.0	212	J Bio Chem 279, 28202 (2004)
3fb4	47.6	7.40	Adenylate kinase	2.00	*Jeotgalibacillus Marinus*	18.0	216	J Bio Chem 279, 28202 (2004)
1s3g	43.4	7.40	Adenylate kinase	2.25	*Bacillus Globisporus*	15.0	217	J Bio Chem 279, 28202 (2004)
1aky	47.7	7.50	Adenylate kinase	1.96	*Saccharomyces Cerevisiae*	28.0	218	Eur J Biochem 231, 405 (1995)
1ank	51.8	7.20	Adenylate kinase	2.00	*Escherichia Coli*	37.0	214	J Biol Chem 266, 23654 (1991)
1zip	74.8	7.20	Adenylate kinase	1.85	*Bacillus Stearothermophilus*	51.0	217	Biochemestry 31, 3038 (1992)
1oa3	49.2	8.00	Cell 12A	1.70	*Hypocrea Schweinitzii*	40.0	217	Protein Sci 12, 848 (2003)
1h8v	54.5	8.00	Cell 12A	1.90	*Trichoderma Reesei*	35.0	217	Protein Sci 12, 2782 (2003)
1oa4	66.8	8.00	Cell 12A	1.50	*Streptomyces sp. 11ag8*	30.0	222	Protein Sci 12, 848 (2003)

1olr	68.7	8.00	Cell 12A	1.20	*Humicola Grisea*	50.0	223	Protein Sci 12, 2872 (2003)
1cec	70.4	7.20	Cell 12A	2.15	*Clostridium Thermocellum*	60.0	331	Biophys Chem 132, 229 (2002)
1csp	53.8	7.00	Cold Shock Protein	2.50	*Bacilllus Subtilis*	37.0	67	J Mol Bio 347, 1063 (2005)
1mjc	57.0	7.00	Cold Shock Protein	2.00	*Escherichia Coli*	37.0	69	Protein Sci 9, 387 (2000), Protein Sci 10, 2028 (2001)
1c9o	76.9	7.00	Cold Shock Protein	1.17	*Bacillus Caldolyticus*	70.0	66	Nat Struct Biol 7, 380 (2000), J Mol Biol 319, 541 (2002)
1bu7	47.0	7.40	Cytochrome p450	1.65	*Bacillus Megaterium*	30.0	455	J Biol Chem 278, 608 (2003)
1oxa	55.1	7.40	Cytochrome p450	2.10	*Saccaropolyspora Erythraea*	31.0	403	J Biol Chem 278, 608 (2003)
1akd	56.0	7.40	Cytochrome p450	1.80	*Pseudomonas putida*	30.0	405	J Biol Chem 278, 608 (2003)
1n97	88.5	7.40	Cytochrome p450	1.80	*Thermus thermophilus*	68.0	385	J Biol Chem 278, 608 (2003)
1f4t	91.0	7.40	Cytochrome p450	1.93	*Solfolobus Solfataricus*	78.0	367	J Biol Chem 278, 608 (2003)
1rgg	49.3	7.00	Ribonuclease	1.40	*Streptomyces aureofaciens*	28.0	96	Biochemestry 37, 16192 (1998), Protein Sci 8, 1843 (1999)
								J Mol Biol 279, 31790 (2003), J Mol Biol 354, 967 (2005)
9rnt	50.9	7.00	Ribonuclease	1.50	*Aspergillus Oryzae*	49.0	104	J Bio Chem 264, 11621 (1989), Biochemestry 33, 3312 (1994)
1rnh	53.2	5.50	Ribonuclease	2.00	*Escherichia Coli*	37.0	155	Eur J Biochem 220, 663 (1994)
1rbn	63.6	7.10	Ribonuclease	2.10	*Bos Taurus*	38.0	123	Biochemestry 45, 10795 (2009)
2ehg	102.0	7.00	Ribonuclease	1.60	*Sulfolobus tokodaii*	80.0	149	PLoS ONE 6, e16226 (2011)
