# Supplementary material for: Stability Curve Prediction of Homologous Proteins Using Temperature-Dependent Statistical Potentials
Source: PLoS Comput Biol. 2014 Jul 17;10(7):e1003689. doi: 10.1371/journal.pcbi.1003689 (PMC4102405; doi:10.1371/journal.pcbi.1003689)
Supplement: Table S2 — Predicted and experimental values of the thermodynamic and thermal parameters for the set of 45 proteins. (PDF) [file pcbi.1003689.s002.pdf]

**Protein**<br>PDB code	$T_m^{\rm exp}$<br>(°C)	$T_m^{\rm pre}$<br>(°C)	$\Delta C_p^{\rm exp}$<br>(kcal/(mol °C)	$\Delta C_p^{\rm pre}$<br>(kcal/(mol °C)	$\Delta G_{25}^{\rm exp}$<br>kcal/mol	$\Delta G_{25}^{\rm pre}$<br>kcal/mol
1aqh	43.7	50.3	8.5	13.0	9.1	12.8
1ppi	65.6	51.2	-	13.1	-	16.9
1jae	65.9	70.5	-	10.5	-	18.0
1smd	70.3	70.2	-	10.6	-	18.3
1am7	52.3	54.4	-	2.6	-	5.8
2lzm	64.8	83.0	2.0	1.5	10.3	5.5
1lz1	64.9	64.8	-	1.7	-	5.1
4lyz	74.8	56.0	1.6	3.4	7.2	7.2
2fal	52.0	56.0	-	2.5	-	4.2
1ymb	78.3	86.8	-	1.4	-	3.7
1bvc	82.2	75.2	2.8	1.7	15.4	5.1
1blc	41.6	54.7	-	2.6	-	9.3
1ke4	54.6	51.0	-	2.5	-	8.4
4blm	66.0	62.8	3.8	2.6	7.1	11.2
1bmc	51.0	40.0	-	2.8	-	5.2
1hml	39.5	53.4	1.1	1.9	-	5.3
1hfz	56.2	60.8	1.4	0.8	4.1	2.8
1hmk	70.8	52.3	-	0.7	-	2.7
2vh7	53.8	71.4	1.5	1.2	4.2	4.6
2bjd	100.8	81.9	1.1	2.0	12.0	9.5
1v3z	111.5	112.9	-	1.4	-	9.6
1p3j	47.6	63.3	-	1.9	-	10.1
3fb4	47.6	49.2	-	1.8	-	7.9
1s3g	43.4	43.6	-	1.9	-	6.3
1aky	47.7	42.0	2.0	1.8	4.1	5.9
1ank	51.8	38.0	-	1.5	-	4.8
1zip	74.8	74.7	-	1.6	-	14.2
1oa3	49.2	48.1	-	2.5	-	6.5
1h8v	54.5	50.3	-	2.9	-	8.1
1oa4	66.8	66.7	-	3.9	-	10.7
1olr	68.7	59.8	-	2.9	-	7.6
1cec	70.4	71.4	-	3.0	-	10.3
1csp	53.8	56.3	1.0	1.1	2.8	2.3
1mjc	57.0	67.5	0.8	1.0	3.0	3.0
1c9o	76.9	63.7	-	1.2	-	4.6
1bu7	47.0	82.1	-	0.9	-	2.5
1oxa	55.1	63.1	-	1.5	-	2.2
1akd	56.0	70.1	1.4	0.8	3.0	1.8
1n97	88.5	56.5	1.1	2.5	5.7	2.2
1f4t	91.2	64.7	-	1.3	-	2.0
1rgg	49.3	45.1	1.5	1.5	5.5	2.4
9rnt	50.9	60.2	1.6	1.0	6.1	3.1
1rnh	53.2	44.5	-	1.5	-	2.2
1rbn	63.6	39.2	1.3	1.9	10.3	2.2
2ehg	102.0	127.3	-	0.7		4.8

Table S 2. Predicted and experimental values of the thermodynamic and thermal parameters for the set of 45 proteins.
