# Supplementary material for: Stability Curve Prediction of Homologous Proteins Using Temperature-Dependent Statistical Potentials
Source: PLoS Comput Biol. 2014 Jul 17;10(7):e1003689. doi: 10.1371/journal.pcbi.1003689 (PMC4102405; doi:10.1371/journal.pcbi.1003689)
Supplement: Table S3 — Analytic expression of the predicted stability curves (in kcal/mol) for the set of 45 proteins. (PDF) [file pcbi.1003689.s003.pdf]

**Protein**<br>PDB code	$\Delta G(T)$<br>kcal/mol
1aqh	3860.99 - 86.8982 T + 12.9713 T Log[T]
1ppi	3866.36 - 89.5051 T + 13.1163 T Log[1.14269 T]
1jae	3212.63 - 71.7649 T + 10.4746 T Log[1.12665 T]
1smd	3239.23 - 72.4588 T + 10.5722 T Log[1.13045 T]
1am7	740.954 - 17.6867 T + 2.58129 T Log[1.20195 T]
2lzm	447.878 - 10.2061 T + 1.47989 T Log[1.18706 T]
1lz1	497.429 - 11.7137 T + 1.7046 T Log[1.20381 T]
4lyz	992.4 - 23.4861 T + 3.41658 T Log[1.21555 T]
2fal	751.643 - 17.189 T + 2.50954 T Log[1.15221 T]
1ymb	410.117 - 9.42587 T + 1.37269 T Log[1.17118 T]
1bvc	524.843 - 11.7968 T + 1.71343 T Log[1.16176 T]
1blc	716.342 - 18.0326 T + 2.62193 T Log[1.28633 T]
1ke4	684.089 - 17.4128 T + 2.5406 T Log[1.27381 T]
4blm	725.826 - 17.9924 T + 2.61018 T Log[1.28217 T]
1bmc	750.606 - 19.2861 T + 2.81409 T Log[1.29056 T]
1hml	622.629 - 14.8865 T + 2.19367 T Log[1.13704 T]
1hfz	272.453 - 6.45412 T + 0.947868 T Log[1.14734 T]
1hmk	232.416 - 5.77366 T + 0.850859 T Log[1.17494 T]
2vh7	351.312 - 8.25193 T + 1.20404 T Log[1.1788 T]
2bjd	585.737 - 13.7018 T + 1.98576 T Log[1.21784 T]
1v3z	443.563 - 9.95537 T + 1.43565 T Log[1.19505 T]
1p3j	524.985 - 13.3849 T + 1.93869 T Log[1.32416 T]
3fb4	639.886 - 16.5572 T + 2.40313 T Log[1.33407 T]
1s3g	667. - 17.3464 T + 2.52038 T Log[1.335 T]
1aky	645.189 - 16.9039 T + 2.45787 T Log[1.33847 T]
1ank	667. - 17.6254 T + 2.56485 T Log[1.34432 T]
1zip	596. - 14.9906 T + 2.16218 T Log[1.33502 T]
1oa3	719.211 - 17.195 T + 2.50838 T Log[1.20521 T]
1h8v	820.577 - 19.5571 T + 2.85048 T Log[1.20689 T]
1oa4	1128.04 - 26.642 T + 3.88385 T Log[1.19568 T]
1olr	839.648 - 19.8894 T + 2.89999 T Log[1.19833 T]
1cec	862.042 - 20.6886 T + 3.01991 T Log[1.20239 T]
1csp	309.138 - 7.3005 T + 1.06576 T Log[1.18791 T]
1mjc	288.612 - 6.72252 T + 0.98012 T Log[1.17757 T]
1c9o	334.18 - 8.10868 T + 1.18453 T Log[1.20768 T]
1bu7	281.869 - 6.23575 T + 0.909218 T Log[1.11956 T]
1oxa	461.178 - 10.4542 T + 1.52719 T Log[1.13786 T]
1akd	252.281 - 5.74163 T + 0.839486 T Log[1.13374 T]
1n97	728.244 - 16.9065 T + 2.47019 T Log[1.16443 T]
1f4t	393.736 - 8.9814 T + 1.30875 T Log[1.1613 T]
1rgg	519.34 - 12.382 T + 1.81022 T Log[1.19216 T]
9rnt	343.652 - 8.11141 T + 1.1846 T Log[1.1829 T]
1rnh	504.735 - 12.0398 T + 1.75963 T Log[1.19517 T]
1rbn	666.692 - 16.0392 T + 2.34435 T Log[1.20578 T]
2ehg	279.16 - 6.01699 T + 0.865223 T Log[1.16887 T]

Table S 3. Analytic expression of the predicted stability curves (in kcal/mol) for the set of 45 proteins.
